# Platelet mitochondria, a potent immune mediator in neurological diseases

**DOI:** 10.3389/fphys.2023.1210509

**Published:** 2023-09-01

**Authors:** Yan Ma, Qian Jiang, Bingxin Yang, Xiaoyu Hu, Gang Shen, Wei Shen, Jing Xu

**Affiliations:** ^1^ Transfusion Research Department, Wuhan Blood Center, Wuhan, Hubei, China; ^2^ Institute of Blood Transfusion of Hubei Province, Wuhan Blood Center, Wuhan, Hubei, China; ^3^ Wuhan National Laboratory for Optoelectronics and School of Physics, Huazhong University of Science and Technology, Wuhan, China; ^4^ Wuhan Puai Hospital, Tongji Medical College, Huazhong University of Science and Technology, Wuhan, Hubei, China; ^5^ Wuhan Blood Center, Wuhan, Hubei, China

**Keywords:** platelet, neuroinflammation, mitochondria, transplantation, central nervous system

## Abstract

Dysfunction of the immune response is regarded as a prominent feature of neurological diseases, including neurodegenerative diseases, malignant tumors, acute neurotraumatic insult, and cerebral ischemic/hemorrhagic diseases. Platelets play a fundamental role in normal hemostasis and thrombosis. Beyond those normal functions, platelets are hyperactivated and contribute crucially to inflammation and immune responses in the central nervous system (CNS). Mitochondria are pivotal organelles in platelets and are responsible for generating most of the ATP that is used for platelet activation and aggregation (clumping). Notably, platelet mitochondria show marked morphological and functional alterations under heightened inflammatory/oxidative stimulation. Mitochondrial dysfunction not only leads to platelet damage and apoptosis but also further aggravates immune responses. Improving mitochondrial function is hopefully an effective strategy for treating neurological diseases. In this review, the authors discuss the immunomodulatory roles of platelet-derived mitochondria (PLT-mitos) in neurological diseases and summarize the neuroprotective effects of platelet mitochondria transplantation.

## 1 Introduction

Platelets, which are small blood cells involved in hemostasis and thrombosis, have been found to initiate immune responses (Koupenova et al., 2022). In addition to their crucial role in blood clotting, platelets also contain a range of innate immune receptors and signaling molecules, which can detect pathogen-associated molecular patterns (PAMPs) and damage-associated molecular patterns (DAMPs) (Dib et al., 2020; Cognasse et al., 2019). Thus, platelets respond rapidly to infection or tissue injury and mount a protective or immune response (Kim et al., 2018; Mauler et al., 2019). Following activation, platelets produce various immune mediators, including cytokines, chemokines, and antimicrobial peptides, and form platelet-neutrophil and platelet-monocyte aggregates that can amplify the immune response (Hottz et al., 2014; Hottz et al., 2020; Mandel et al., 2022). Dysfunction of platelets is regarded as a hallmark of neurological diseases, such as neurodegenerative diseases, malignant cerebral tumors, acute neurotraumatic insult, and cerebral ischemic/hemorrhagic diseases, etc. (Mendoza-Sotelo et al., 2010; Carrizzo et al., 2014; Fišar et al., 2016; Prodan et al., 2016; Perez et al., 2018; Hishizawa et al., 2019; Dziedzic et al., 2020; Gong et al., 2021; Kim HK. et al., 2022; Sloan et al., 2022; Wang et al., 2023a) **(** Table 1 **)**. Previous studies have found that activated platelets in the pathological environment of the brain cause the overactivation of microglia and astrocytes (Bhat et al., 2017). For instance, platelets obtained from hypertensive rats showed significantly higher levels of soluble CD40 ligand and caused more pronounced activation of glial cells (astrocytes and microglia), triggering the nuclear factor kappa B (NF-κB) and mitogen-activated protein kinase (MAPK) inflammatory signaling pathways, which culminated in neuronal injury and elevated levels of apoptotic cells (Bhat et al., 2017). However, platelets and platelet lysates also exert neuroprotective effects due to the neurotrophic, antioxidative, and anti-inflammatory functions of the platelet proteome and platelet-derived extracellular vesicles (EVs) (Nebie et al., 2021; Burnouf and Walker, 2022). A better understanding of the complex roles of platelets will hopefully provide effective therapies for neurological disorders.

**TABLE 1:** Platelet dysfunctions in neurological disorders.

CNS diseases	Key findings	References
Alzheimer’s disease	Platelets from AD patients have decreased respiration rates and mitochondrial dysfunctions	[9]
Parkinson’s disease	Reduced platelet 5-HT content is associated with rest tremor in Parkinson’s disease	[10]
Huntington’s disease	Platelets of HD patients have reduced eNOS phosphorylation (Ser(1177)) and activity	[11]
Multiple sclerosis	Platelets of MS patients show mitochondrial membrane potential disruption and elevated production of ROS.	[12]
Amyotrophic lateral sclerosis	TDP-43 concentration in platelets was significantly higher in patients with ALS.	[13]
Major depression disease	Platelets from depressed patients had a greater proportion of dendritic forms and altered immunolocalization of P-selectin	[14]
glioma/glioblastoma multiforme	Glioma stem cells (GSCs) produce thrombin and contribute to platelet activation	[15]
stroke	Platelet to lymphocyte ratio (PLR) was associated with post-thrombolysis early neurological deterioration (END) may predict post-thrombolysis END.	[16]
traumatic brain injury	Coated-platelet levels are elevated in patients with combat-related mild TBI.	[17]
Cerebral aneurysm	Lower platelet-to-neutrophil ratio and platelet-to-white-blood-cell ratio are associated with ruptured intracranial aneurysm and a higher PHASES score	[18]
Subarachnoid hemorrhage	Platelets from aneurysmal subarachnoid hemorrhage patients showed prolonged increases in activation and aggregation	[19]

**FIGURE 1:**
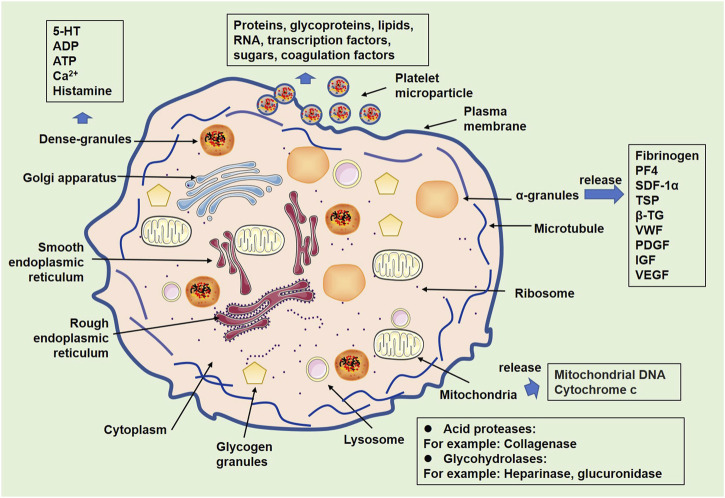
Organelles of platelets and related products. Following activation, platelets show alterations of organelles. Some organelles, such as *α*-granules, dense granules, lysosomes, and mitochondria, contain multiple substances that can be released from activated platelets.

Platelets are small subcellular fragments formed from the cytoplasm of bone marrow megakaryocytes (Machlus and Italiano, 2013). Although platelets lack a nucleus and most organelles found in typical cells, they do contain a few specialized subcellular structures, such as organelles, including mitochondria, lysosomes, and peroxisomes (Thon and Italiano, 2012) (Figure 1). *α*-granules are membrane-bound organelles that contain proteins such as fibrinogen, von Willebrand factor (VWF), and platelet-derived growth factor (PDGF). These protein molecules are released when platelets become activated and play a critical role in hemostasis, wound healing, and angiogenesis (Smith, 2022). For dense granules, small organelles contain molecules such as serotonin, ADP, and ATP. When platelets are activated, these molecules are released to promote platelet aggregation and vasoconstriction (Golebiewska and Poole, 2015). In addition, platelets contain a small number of mitochondria, which provide the energy needed for platelet function (Misztal et al., 2014). Platelet-derived mitochondria, which are regarded as the main source of circulating mitochondria (freeMitos) (Zhao et al., 2017a; Stephens et al., 2020), exert wide biological functions, such as responses of CD4^+^ T cells (Yu et al., 2020), and metabolic remodeling of mesenchymal stem cells (MSCs) (Levoux et al., 2021). Thus, platelets have pivotal roles in many physiological processes, such as angiogenesis (Hayon et al., 2012), innate immunity (Jiang et al., 2022), adaptive immunity (Koupenova et al., 2018), neurogenesis (Delgado et al., 2021), wound healing, and tissue regeneration (Fan Y. et al., 2020). Moreover, increasing evidence has supported that platelets are involved in mediating the pathological processes of central nervous system (CNS) diseases (Mezger et al., 2015).

**FIGURE 2:**
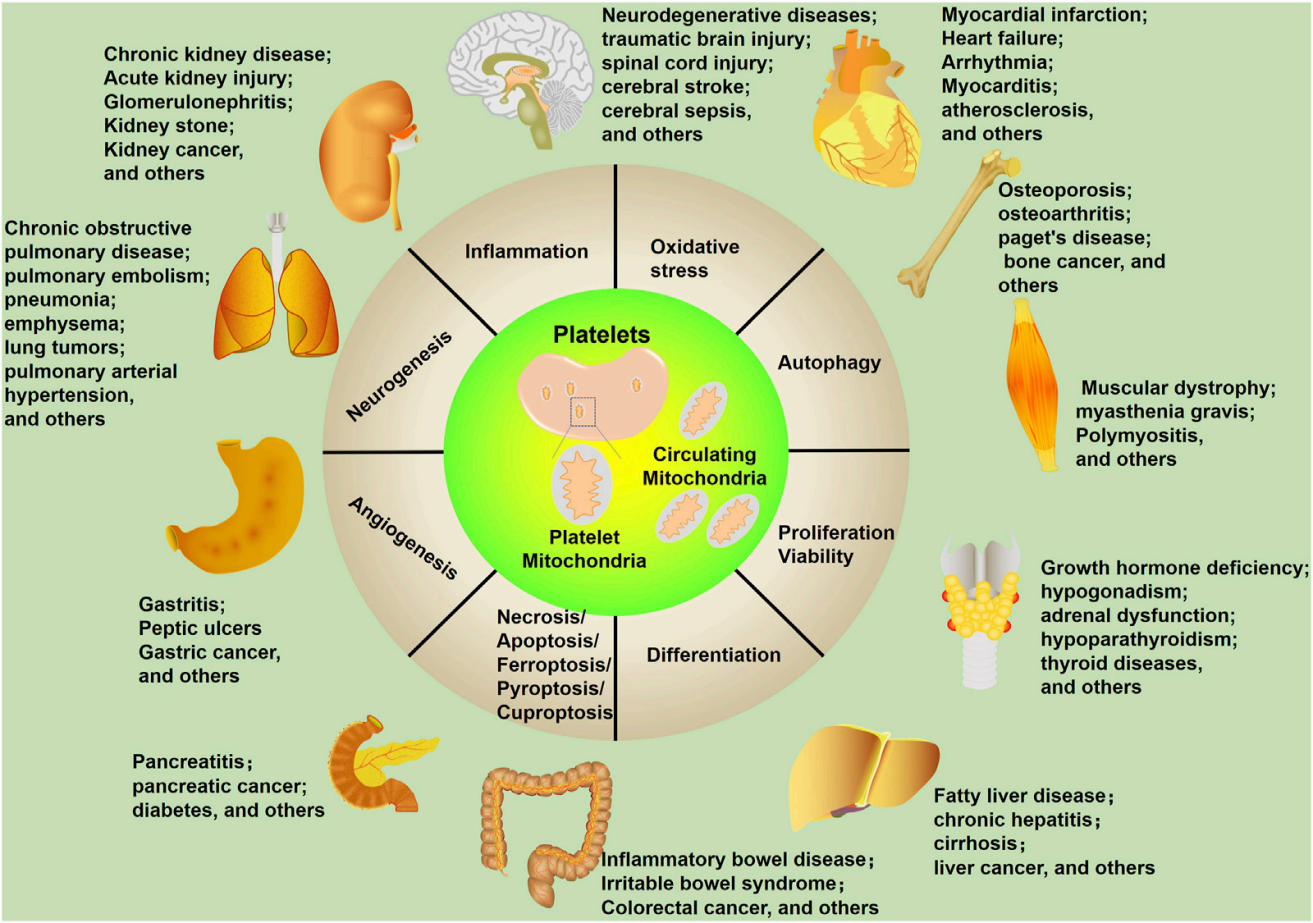
Mitochondrial biological functions in human diseases. Mitochondria affect multiple cellular processes, including inflammation, oxidative stress, autophagy, proliferation/viability, differentiation, necrosis/apoptosis/ferroptosis/pyroptosis/cuproptosis, angiogenesis, and neurogenesis. These cellular processes are closely related to human diseases, including neurological diseases.

Mitochondria function as the “power plant” of eukaryotic cells and play an important role in energy metabolism (Friedman and Nunnari, 2014). Beyond this basic function, mitochondria are regarded as essential mediators of multiple biological processes and aggravate the development of diseases (Figure 2). In this review, we will summarize the roles of platelet-derived vesicles, as well as platelet mitochondria in neurological disorders. In addition, platelet mitochondria transplantation shows high potential in the treatment of those diseases by acting as a potent mediator of cell metabolism, apoptosis, inflammatory reactions, and oxidative stress. We will also discuss the advantages and limitations of the use of platelet mitochondria transplantation.

## 2 The alterations of platelet mitochondria (PLT-mitos) in CNS diseases

Mitochondrial alterations, such as mitochondrial morphology changes, impaired respiratory chain function, calcium homeostasis disruption, and altered mitochondrial DNA (mtDNA), are hallmarks of CNS disorders (Kilbaugh et al., 2015; Monzio Compagnoni et al., 2020; Ding et al., 2023). Platelets play a crucial role in CNS diseases, and manifold functions of platelets have been found, far more than hemostasis and thrombosis (Figure 2). Platelets play a role in synaptic plasticity, memory, and learning, and those activated by physical activity encourage the development of neurons in certain regions of the brain. Additionally, platelets play a part in the immune response by altering their surface protein profile and releasing pro- and anti-inflammatory substances (Koupenova et al., 2018; Rawish et al., 2020; Ferrer-Raventós and Beyer, 2021). Notably, PLT-mitos also show significant functional alterations in CNS diseases (Bronstein et al., 2015; Donner et al., 2021). Thus, evaluating the functions of PLT-mitos helps the diagnosis and severity evaluation of those diseases.

### 2.1 Cerebrovascular diseases and PLT-mitos dysfunction

Cerebrovascular diseases, mainly including stroke, aneurysms, arteriovenous malformations (AVMs), transient ischemic attack (TIA), and moyamoya disease, are accompanied by platelet dysfunctions (Maki et al., 1981; Sutherland et al., 1988; Bigalke et al., 2010; He et al., 2019; Rosińska et al., 2019). Mitochondrial dysfunction causes mitochondrial oxidative phosphorylation and cellular bioenergetic stress, and this is one of the critical elements in the development of delayed neuronal death, a phenomenon that follows cerebral ischemia in the brain with full force (Martorell-Riera et al., 2014). Mitochondrial dynamics evaluation is an adjunctive approach for neuroprotective time window extension after ischemic stroke (Shen et al., 2021).

Alterations in platelet mitochondria in cerebrovascular diseases have been revealed by previous studies. In stroke patients, fewer alpha granules and mitochondria were found in platelets (Joseph et al., 1989). Aneurysmal subarachnoid hemorrhage (aSAH) is a complex disease that threatens people’s lives due to cerebral vasospasm (CVS) and delayed cerebral ischemia. Cytochrome B (CytB), D-loop, and cytochrome c oxidase subunit-1 (COX-1) are three representative mitochondrial gene fragments, and they all have significant relationships with post-SAH complications, suggesting that mtDNA may directly or indirectly influence post-SAH complications and clinical outcomes (Chaudhry et al., 2019). Baccarelli et al. examined platelet mtDNA methylation in cardiovascular disease patients via bisulfite-PCR pyrosequencing. They found that CVD patients have higher levels of platelet mtDNA methylation than healthy controls, indicating that platelet mtDNA methylation might be a non-invasive and easy-to-obtain marker in CVD (Baccarelli and Byun, 2015).

### 2.2 Neurodegenerative diseases and PLT-mitos dysfunction

Neurodegenerative diseases are characterized by the progressive loss of function and death of neurons in the brain and/or the spinal cord (Chi et al., 2018). Mitochondrial dysfunction has been regarded as a main feature of neurodegenerative diseases, including Alzheimer’s disease (AD) (Sukhorukov et al., 2021), Parkinson’s disease (PD) (Borsche et al., 2021), Huntington’s disease (HD) (Šonský et al., 2021), amyotrophic lateral sclerosis (ALS) (Chen et al., 2022), and multiple sclerosis (MS) (Faissner et al., 2019). Recently, platelets and PLT-mitos have been found to be mediators in neurodegenerative diseases (Wang et al., 2017; Ferrer-Raventós and Beyer, 2021). For instance, platelets are a major source of amyloid-beta (Aβ), which is a key protein involved in the pathogenesis of AD. Increased platelet activation has been observed in AD patients, and platelet-derived Aβ has been shown to promote neuroinflammation and neuronal damage (Li and Liu, 2022). In PD, increased platelet *α*-synuclein (αSyn) is associated with elevated ROS production and mitochondrial dysfunction (Shults et al., 2006). In addition, exogenous αSyn has mild platelet antiaggregating properties *in vitro* by preferentially binding to the outer surface of activated platelets (Acquasaliente et al., 2022). Platelets show abnormal functions in HD, as reflected by the release of angiogenic factors that function in thrombosis, angiogenesis, and vascular hemostasis (Denis et al., 2019). Platelet-derived serotonin levels have a strong relationship with survival in ALS (Dupuis et al., 2010). Mitochondrial dysfunction was observed in platelets and peripheral blood mononuclear cells from the blood of ALS patients, i.e., complex IV activity decline in mononuclear cells and mitochondrial content elevation in platelets (Ehinger et al., 2015). The evidence from the ultrastructure study showed that nonuniformity of matrix, faint cristae, greater lysosomal bodies, and fewer intramitochondrial granules were observed in ALS PLT-mitos (Shrivastava et al., 2011a). Another study suggested that PLT-mitos in ALS patients have perturbance of MMP, mitochondrial depolarization, and elevated apoptosis (Shrivastava et al., 2011b). Platelets of multiple sclerosis (MS) patients have higher mitochondrial aconitase activity and mitochondrial lipid peroxidation, decreased cytochrome c levels, and enhanced mitochondrial SOD1 expression (Iñarrea et al., 2014).

### 2.3 PLT-mitos dysfunction in CNS infection

Patients with CNS infection experience very high morbidity and mortality. Even though physiological and immunological barriers typically limit pathogen entry into the brain parenchyma and retina, some pathogens can overcome these defenses and initiate innate immunity within the CNS (Forrester et al., 2018; Li et al., 2021). In response to the stimulation of pathogens, such as human immunodeficiency virus (HIV) (Ganta and Chaubey, 2019) and coronavirus (SARS-CoV-2) (Saleh et al., 2020), there are obvious mitochondrial dysfunctions, such as changes in morphology, membrane depolarization, mitophagy, mtDNA depletion, and intrinsic apoptosis. During infection, platelet mitochondria also show alterations. For instance, people living with HIV have reduced *ex vivo* platelet reactivity and mean platelet volume. Platelet mtDNA has a positive correlation with both platelet parameters and a negative correlation with the inflammatory marker sCD163 (van der Heijden et al., 2021). Platelets isolated from COVID-19 patients had a reduced procoagulant ability and reduced mitochondrial depolarization and phosphatidylserine exposure under stimulation with thrombin and convulxin (Denorme et al., 2020). Platelet mitochondrial respiratory chain function, oxidative phosphorylation, and endogenous CoQ10 levels were reduced in patients after COVID-19 (Sumbalova et al., 2022). Sepsis is a leading cause of morbidity and mortality and often contributes to acute brain dysfunction (Tong et al., 2021). PLT-mitos membrane depolarization is significantly correlated with the severity of sepsis (Gründler et al., 2014). Thus, evaluating platelet mitochondrial functions might help in the diagnosis of CNS infections.

### 2.4 PLT-mitos dysfunction in psychiatric disturbances

There is evidence to suggest that mitochondrial dysfunction may contribute to the development of psychiatric disorders, such as major depression (Rezin et al., 2009), bipolar disorder (Zvěřová et al., 2019), autism (Abdel-Rahman et al., 2021), and schizophrenia (Rezin et al., 2009). Evaluating mitochondrial functions is helpful in the diagnosis and treatment assessment of psychiatric disturbances. For example, a reduced leukocyte mtDNA copy number was found in schizophrenia (Shivakumar et al., 2020) and bipolar disorder type I (de Sousa et al., 2014). Increased reactive oxygen and nitrogen species and lower levels of key antioxidants are hallmarks of major depression patients. Damage to mitochondria and mtDNA and reduced activity of respiratory chain enzymes and adenosine triphosphate production (Maes et al., 2012). Platelet mitochondria serve as an independent indicator of psychosis. For example, platelet mitochondrial complex I activity shows an increase in high-positive schizophrenic patients and is positively correlated with the Positive and Negative Syndrome Scale scores (Ben-Shachar et al., 2007). Altered nitric oxide (NO) levels, mitochondrial membrane potential (PMMP), and p-selectin expression in platelets were also observed in major depressive disorder patients (Moreno et al., 2013). Another study also supported that there are significant differences in mitochondrial parameters, including citrate synthase (CS) activity, electron transport system (ETS) complex (complexes I, II, and IV) activities, and mitochondrial respiration in blood platelets (Zvěřová et al., 2019).

## 3 Functions of PLT-derived mitochondria (freeMitos and mitoMPs) in CNS diseases

Various CNS diseases, such as neurodegenerative diseases (such as PD, AD, and HD), cerebral stroke, traumatic brain injury (TBI), and spinal cord injury (SCI), are associated with dysfunctions of oxidative stress and inflammatory reactions (Islam, 2017; Khatri et al., 2018; Kumar et al., 2023). PLT-mitos have close relationships with mitochondrial DNA lesions, electron transfer chain impairments, mitochondrial apoptosis, and mitophagy in diverse human diseases (Forrester et al., 2018). Circulating mitochondria released by platelets are generally considered a source of potential DAMPs, promoting inflammation and oxidative stress (Zhang et al., 2010; Forrester et al., 2018; Linge et al., 2018). Extracellular and active mitochondria in the CSF have been detected, and extracellular mitochondria act as a biomarker for the outcome of pathologies such as SAH and delayed cerebral ischemia (Caicedo et al., 2021).

EVs are membrane vesicles released from the cellular plasma membrane (microvesicles or microparticles (MPs)) or endosomal compartments (exosomes) of cells. Following TBI, the injured brains produced cellular microvesicles, which further induced consumptive coagulopathy. Extracellular mitochondria, accounting for 55.2% of these microvesicles, contributed to TBI-induced coagulopathy, oxidative stress, and inflammation by enhancing platelet procoagulant activity (Zhang et al., 2022). Platelets are normally absent in the CNS, while they produce abundant EVs after being activated. The EVs are released into circulation and acquired by vascular endothelial cells through the process of endocytosis (Puhm et al., 2021; Gardin et al., 2022). Platelet-derived EVs exhibit a predictive value for recurrent vascular events in patients after ischemic stroke (Rosińska et al., 2019). Notably, platelets contain enriched mitochondria that may contribute to the local reactive oxygen species pool and remodel phospholipids in the plasma membrane of blood vessels (Aggarwal et al., 2023). Boudreau et al. suggested that respiratory-competent mitochondria are released from activated platelets. Those mitochondria are encapsulated by MPs (mitoMPs) and as freeMitos (Boudreau et al., 2014). Stephens and his colleagues analyzed circulating MPs using flow cytometry and proteomics for identifying the sources of mitoMPs and freeMitos. The results showed that mitochondria-containing MPs that were derived from murine and human beings had positive expressions of CD41 (a platelet marker) and CD144 (the endothelial cell marker), while hematopoietic CD45 labeling was lowly expressed, suggesting that circulating mitochondria mainly originated from platelets, endothelial cells, and leukocytes (Stephens et al., 2020). At the same time, Al Amir Dache Z et al. demonstrated that functional freeMitos are released from resting platelet in a normal physiological state. In addition, normal and tumor cultured cells can also secrete their mitochondria (Al Amir Dache et al., 2020). PLT-mitos share characteristics with bacterial and mitochondrial damage-associated molecular patterns, which are important contributors to sterile inflammation processes (Léger et al., 2022). Therefore, it is believed that freeMitos have the potential for early detection and prognosis of various diseases (Al Amir Dache et al., 2020).

PLT-mitos, whether shuttled by PLT-EVs or as freeMitos, play a role in neurological diseases. For instance, platelet-derived exosomes are significantly elevated in the serum of AD patients (Odaka et al., 2021). Alterations in platelet-derived EVs are also found in other neurodegenerative diseases, such as HD (Denis et al., 2018) and PD (Wang et al., 2023b). In patients with subacute stroke, platelets formed the largest population of vesicles within serial blood samples, and lower levels of platelets vesicles were associated with a worse functional outcome in the first 6 months post-stroke (Jödicke et al., 2021). For stroke patients who underwent the traditional rehabilitation program, the combination of exercise training improved platelet mitochondrial oxidative phosphorylation and electron transport chain, and mitigated plasma myeloperoxidase and interleukin-6 levels (Hsu et al., 2019). Extracellular mitochondria (exMTs) also exert a role in TBI (Zhang et al., 2022). As reviewed by Zhao et al., exMTs are released from injured cerebral cells, endothelial cells, and platelets. These circulating exMTs induce potent procoagulant activity and also aggravate inflammation, oxidative stress, and contribute to secondary tissue injury after the primary traumatic impact (Zhao et al., 2020). Glioblastoma multiforme (GBM) often develops deep venous thrombosis or pulmonary emboli, suggesting that platelets potentially involve in those pathological changes (Riedl and Ay, 2019). Gonzalez-Delgado et al. verified that freeMitos were increased in GBM patients compared with healthy controls. Intravenous delivery of mitochondria resulted in an increased rate of venous thrombosis in a murine model of inferior vena cava stenosis. Mitochondria-induced venous thrombi contain rich neutrophils and more platelets than those in control thrombi. Hence, the authors concluded that mitochondria might play a role in the GBM-induced hypercoagulable state (Gonzalez-Delgado et al., 2023). Overall, freeMitos and mitoMPs in circulation play a pivotal role in neurological diseases. Further investigations are needed to address the specific role of PLT-mitos in CNS diseases.

## 4 Mediators of platelet mitochondria in CNS diseases

**FIGURE 3:**
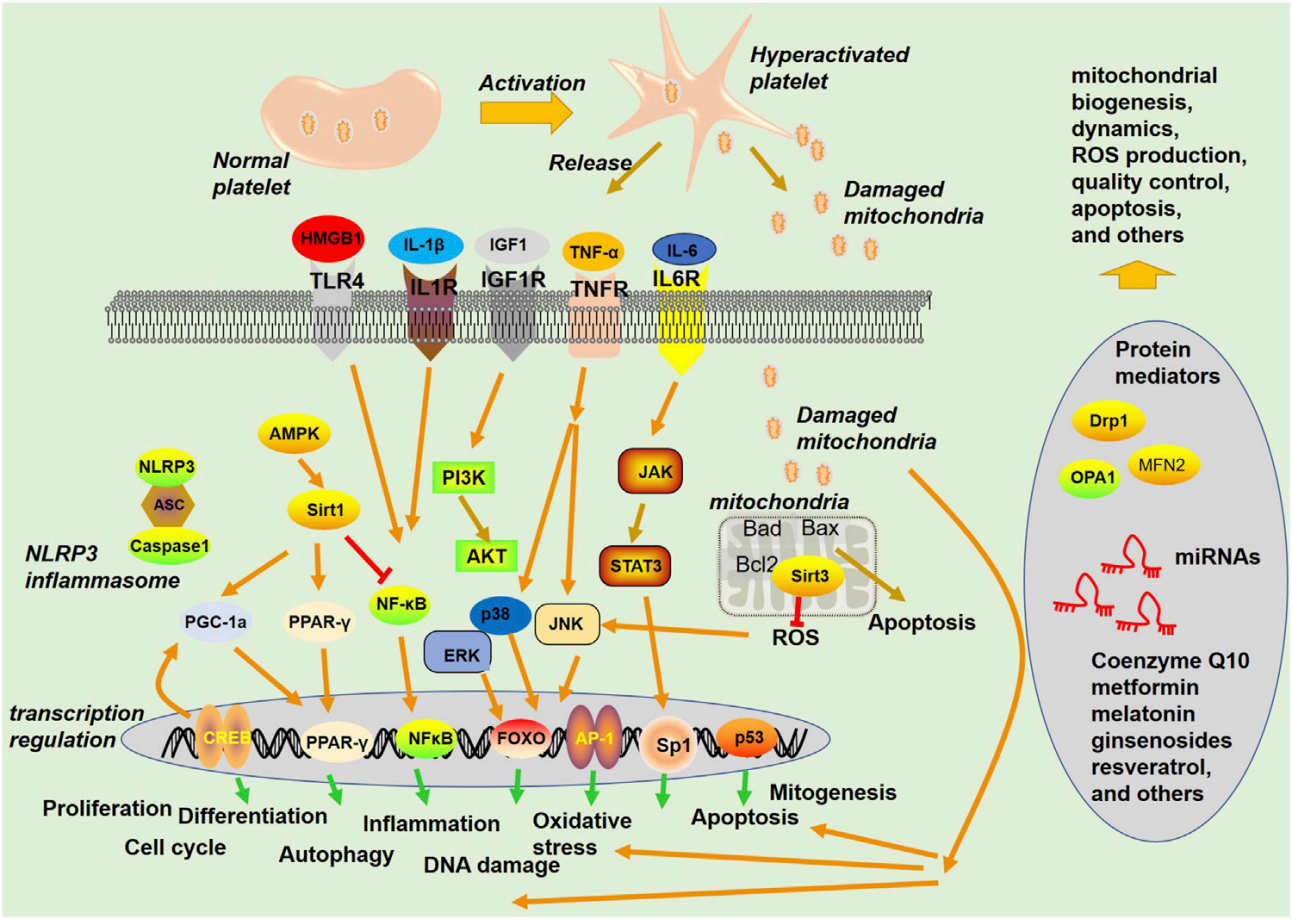
Signaling pathways and proteins involved in mitochondria-mediated functions. Platelets become hyperactivated upon stimuli from blood under a pathological environment, are released from activated platelets, and act as a damage-associated molecular pattern (DAMP) to stimulate the innate immune system. Mitochondria can exert multiple functions, such as regulating apoptosis and ROS production.

The functional integrity of mitochondria is regulated by a complex network of molecular mechanisms that control mitochondrial biogenesis, dynamics, quality control, and communication with other cellular compartments. At the transcriptional level, the transcription of mitochondrial genes is regulated by mitochondrial or nuclear transcription factors that bind to the promoters of the mitochondrial genome (Gustafsson et al., 2016). Alterations in the expression of these factors can impact the expression of mitochondrial genes and consequently disrupt mitochondrial function (Bonekamp et al., 2020). In addition, posttranscriptional regulation mechanisms, such as RNA splicing, can modulate the expression of mitochondrial genes, and their deregulation can contribute to mitochondrial dysfunction (Tsuboi et al., 2020). Moreover, proteins that are essential for mitochondrial function, such as enzymes, channels, and transporters, are synthesized in the cytoplasm and subsequently transported into the mitochondria (Busch et al., 2023) (Figure 3).

### 4.1 Proteins affecting platelet mitochondrial functions

Many molecules are responsible for mitochondrial functions. The imbalance of mitochondrial dynamics results from dysfunctions in key components of the fission and fusion machinery, including dynamin-related protein 1 (Drp1), mitofusins 2 (MFN2), and optic atrophy protein 1 (OPA1) (Yapa et al., 2021). In addition to karyocytes, the functions of platelet mitochondria are also affected by key mediators. Improving platelet mitochondrial functions through control of mitochondrial oxidative stress production or inhibition of respiratory complexes shows good potential in the treatment of platelet activation-related diseases (Fuentes et al., 2019). For example, enhanced reactivity of platelets from polycystic ovary syndrome (PCOS) patients has been demonstrated and is associated with the impaired activation of AMP-activated kinase (AMPK). An increased phosphorylated level of Drp-1 on Ser616 was found, while that on Ser637 was decreased. Metformin treatment recovered mitochondrial function in both platelets and megakaryocytes. Thus, metformin reduced platelet hyperactivity (Randriamboavonjy et al., 2015). A recent study verified that during thrombopoiesis, Mdivi-1-mediated inhibition of Drp1-dependent mitochondrial fission of mature megakaryocytes supported a tubular mitochondrial network and mitigated mitochondrial ROS (mtROS) levels and intermediate MK proportions, whereas enhancing Drp1 activity genetically had the opposite effects (Poirault-Chassac et al., 2021). Overall, the above studies suggest that it makes much sense in mediating platelet mitochondrial functions.

### 4.2 Pharmaceuticals regulate platelet mitochondrial functions in CNS diseases

Considering the fundamental roles of mitochondria in disease progression, many pharmaceuticals or agents have been developed to treat diseases by regulating mitochondrial functions (Bhatti et al., 2017; Bonora et al., 2019; Supinski et al., 2020). Among those drugs, mitochondria-targeted antioxidants have attracted increasing attention. Coenzyme Q10 (CoQ10) is an antioxidant and a key component of the electron transport chain in mitochondria. It has been used to improve mitochondrial function in various diseases, including heart failure, PD, and mitochondrial myopathy (Rauchová, 2021; Testai et al., 2021). Other antioxidants with mitochondrial regulatory effects, such as *α*-lipoic acid (Karalis et al., 2021), N-acetylcysteine (Patel et al., 2014), ubiquinone (MitoQ) (Maiti et al., 2018), and vitamin E (Villalón-García et al., 2022), have all exhibited marked neuroprotective effects in CNS disorders. Since AMPK (Watters et al., 2020), Sirt1 (Yang et al., 2017), Peroxisome proliferator-activated receptor (PPAR)γ (Pizcueta et al., 2023), and PGC-1α (Han et al., 2021) are crucial in maintaining normal functions and preventing damage to mitochondria, their activators or agonists have been used as effective mediators of mitochondria in the CNS (Xu et al., 2021; Yeh et al., 2021).

Interestingly, many conventional pharmaceuticals in the clinic have been found to mediate mitochondrial functions. For instance, metformin is a widely used diabetes medication that has been shown to have beneficial effects on mitochondrial functions. It activates AMPK, a key regulator of mitochondrial biogenesis and metabolism, and increases mitochondrial fatty acid oxidation in PD and brain injury postcardiac arrest (Mor et al., 2020; Shoaib et al., 2022). Melatonin, a pineal hormone for physiologic processes and a guardian of body homeostatic balance, has been found to have several functions related to mitochondrial health, including scavenging free radicals generated during mitochondrial respiration, mitochondrial biogenesis, mitochondrial membrane stabilization, and regulation of mitochondrial metabolism and dynamics (Chitimus et al., 2020; Skemiene et al., 2020; Arinno et al., 2021). Melatonin has been used for treating CNS disorders by maintaining mitochondrial health and function (Demaré et al., 2021).

Notably, pharmaceuticals that have anti-platelet effects or agents used for platelet storage have a role in mediating mitochondrial functions (Amorini et al., 2013; Fuentes et al., 2018). Several antiplatelet agents, such as aspirin, enhance mitochondrial biogenesis by mediating Sirtuin1/PGC-1α (Freixer et al., 2021). However, high-dose clopidogrel inhibits mitochondrial respiration by reducing mitochondrial oxidative phosphorylation (Zahno et al., 2013). During platelet storage, supplementation with resveratrol and cytochrome c maintained platelet aggregation, morphology, intracellular ROS, and mitochondrial function (Ekaney et al., 2022). Trehalose is an ideal agent for platelet storage because it improves the apoptosis, viability, and survival rate of platelets at cold temperatures (Baghdadi et al., 2022). Moreover, trehalose improved the desiccation tolerance of mammalian mitochondria (Fan Rui-Feng et al., 2020) and inhibited the mitochondrial apoptotic signaling pathway in cadmium-induced kidney injury (Liu et al., 2005), suggesting that trehalose has a role in maintaining platelets by mediating mitochondrial function.

Improving mitochondrial functions has been regarded as a promising strategy for treating brain injury following primary insult in cerebrovascular diseases (An et al., 2021). Many pharmaceuticals or chemical agents, such as metformin (Xin et al., 2016; Leech et al., 2020), curcumin (Bavarsad et al., 2019), and rapamycin (Li et al., 2014; Śledź et al., 2020), have dual roles in cerebrovascular diseases and platelet activation by protecting mitochondrial functions. Therefore, strategies targeting platelet mitochondrial dysfunction could be an effective approach to attenuate neurological damage resulting from cerebrovascular diseases.

## 5 Platelet mitochondria transplantation in CNS diseases

Studies have revealed that mitochondria are not limited to host cells and can actively move between cells through extracellular vesicles or nanotubes, and the transport of mitochondria plays a crucial role in metabolic homeostasis, the immune response, and stress signaling (Wang ZH. et al., 2022). Replacing damaged or dysfunctional mitochondria with exogenous healthy mitochondria, also called mitochondrial transplantation, has shown promising results (Gollihue and Rabchevsky, 2017). In particular, animal experiments have suggested that mitochondrial transfer is an effective strategy for treating several CNS disorders, such as SCI (Fang et al., 2021), TBI (Bamshad et al., 2023), cerebral stroke (Liu et al., 2019), PD (Moradi Vastegani et al., 2023), and depression (Javani et al., 2022). In 2021, a clinical trial (NCT04998357) was started to confirm the safety of autologous mitochondrial transplantation during brain ischemia.

Recently, increasing evidence has shown that platelet-extracted mitochondria transplantation plays a role in several cellular dysfunctions and diseases (Popov, 2021). Functionally, platelets and their released mitochondria exhibit markers associated with immune tolerance and regulate immune cell proliferation and functions. Platelet mtDNA also expressed embryonic stem cell- and pancreatic islet *ß*-cell-associated markers. Platelet-releasing mitochondria can migrate to pancreatic islets and be taken up by islet *ß* cells, leading to the proliferation and enhancement of islet *ß*-cell functions (Zhao et al., 2017b). Platelet mitochondria can be internalized into human dermal fibroblasts, increase cell proliferation, promote closure of the wound gap, and relieve intracellular and mitochondrial ROS production, thus enhancing wound healing in a cellular model (Kim S. et al., 2022). In primary cardiomyocytes, platelet-derived mitochondria transplantation increased mitochondrial membrane potential and greater ATP synthase activity and citrate synthase activity (Lin et al., 2023). In SH-SY5Y cells subjected to hypoxia/reoxygenation (HR) injury, platelet-derived mitochondria transplantation significantly mitigated mitochondrial malfunction and the mitochondrial apoptotic pathway. This study suggests that platelets may serve as readily available sources of donor mitochondria that afford therapeutic benefits against ischemia/reperfusion injury (Shi et al., 2021). In a diabetes-associated cognitive impairment (DACI) mouse model, platelet-derived mitochondria were internalized into hippocampal neurons 24 h after intracerebroventricular injection. One month later, the cognitive impairment of DACI mice was relieved. Platelet-derived mitochondria also increased mitochondrial number, improved mitochondrial function, suppressed neuronal oxidative stress and apoptosis, and inhibited the accumulation of Aβ and Tau in the hippocampus (Ma et al., 2020). Overall, platelet mitochondria therapy is a promising tool for promoting tissue repair and regeneration. More research is needed to better understand its mechanisms of action and to optimize its use in clinical settings.

## 6 Future prospects

Platelet mitochondrial dysfunctions have been uncovered in multiple CNS diseases, which can be caused by genetic mutations in mitochondrial and nuclear DNA, as well as by the pathological environment of a certain disease. Platelet-based liquid biopsy has aroused increasing attention in disease diagnosis, especially in cancer diagnosis (Wang L. et al., 2022). However, there are limited studies available regarding the discovery of platelet-mitos in the diagnosis of CNS disorders. Platelet and mitochondrial dynamics have rapid reactions to the alterations of diseases. Mitochondrial behaviors and changes in size, shape, and location within the cell occur in response to different physiological and pathological cues. These behaviors include mitochondrial fission, fusion, movement, distribution, and degradation, and the balance between them is critical for maintaining mitochondrial function and cellular homeostasis. Platelet mtDNA contains genetic information about diseases, and this might provide a more precise diagnosis of CNS diseases. Second, since platelet mitochondria also function as mediators in the initiation and progression of CNS diseases, mediators and drugs that regulate platelet mitochondrial dysfunction may have more potential in treating those diseases. Third, as increasing evidence has supported that platelet mitochondria transplantation has significant effects in treating cellular dysfunction and diseases, PLT-mitos transfer is a prospective strategy for the treatment of CNS diseases. In comparison to other sources of mitochondria, such as autologous muscle and mesenchymal stem cells, platelets are more accessible and regenerative. Moreover, studies should be performed to improve the isolation method of functional mitochondria from platelets (Vernerova et al., 2021).

## 7 Conclusion

Accumulated studies have revealed that platelet mitochondrial alterations have a close relationship with the development of CNS diseases, and evaluating platelet mitochondrial functions helps the prediction of treatment outcomes. Further studies will elucidate the role of platelet-mitos as a rapid and precise method for the diagnostic and therapeutic evaluation of CNS disorders. Moreover, methods for improving platelet-mitos isolation and experiments on the functions of platelet-mitos transplantation in CNS diseases are necessary.
